# Incremental Prognostic Value of Neutrophil–Lymphocyte Ratio in Normotensive Patients with Intermediate-Risk Acute Pulmonary Embolism

**DOI:** 10.3390/jcm15072661

**Published:** 2026-03-31

**Authors:** Rodica Lucia Avram, Elena Barbu, Monica Mariana Baluta, Irina Toma, Anna Maria Andronescu, Alexandru Cristian Nechita

**Affiliations:** 1 “Carol Davila” University of Medicine and Pharmacy, 020021 Bucharest, Romania; rodica.ploesteanu@umfcd.ro (R.L.A.); monicabaluta@yahoo.com (M.M.B.); anna.andronescu@gmail.com (A.M.A.); cardionechita@yahoo.com (A.C.N.); 2 “Sf. Pantelimon” Emergency Hospital, 021659 Bucharest, Romania; irinatoma05@yahoo.com; 3 Elias University Emergency Hospital, 011461 Bucharest, Romania

**Keywords:** acute pulmonary embolism, intermediate risk, neutrophil–lymphocyte ratio, risk stratification, in-hospital mortality

## Abstract

**Background**: Risk stratification in normotensive patients with acute pulmonary embolism (APE) remains challenging, particularly within the heterogeneous intermediate-risk category. The neutrophil–lymphocyte ratio (NLR), an accessible marker of systemic inflammation, has emerged as a potential prognostic indicator. We aimed to evaluate the additional prognostic value of NLR in normotensive patients with intermediate-risk APE. **Methods**: We conducted a retrospective analysis of 402 consecutive normotensive patients with imaging-confirmed APE. Patients were classified according to European Society of Cardiology (ESC) risk stratification. The primary endpoint was in-hospital mortality. Laboratory, clinical, and echocardiographic parameters were analyzed. Logistic regression was performed to identify independent predictors of in-hospital death. Discriminative performance was assessed using receiver operating characteristic (ROC) analysis. **Results**: In-hospital mortality occurred in 13.9% of patients. NLR values were significantly higher among non-survivors. On multivariable logistic regression, NLR retained independent prognostic significance, with values exceeding one standard deviation (5.66) indicating an approximately twofold higher risk of in-hospital mortality. Echocardiographic right ventricular dysfunction and troponin I levels were not independent predictors in the adjusted model. NLR demonstrated modest discriminative ability (AUC = 0.650). A cut-off value of 5.49 provided the best balance between sensitivity and specificity. The addition of NLR to ESC risk stratification improved the identification of patients at higher risk within the intermediate category. **Conclusions**: In normotensive patients with intermediate-risk APE, NLR represents a simple and readily available biomarker with independent prognostic value. Its incorporation into current risk stratification algorithms may enhance early identification of patients at increased risk of in-hospital mortality. Prospective validation studies are warranted.

## 1. Introduction

Acute pulmonary embolism (APE) is the third most common cause of cardiovascular mortality worldwide [1], following myocardial infarction and cerebrovascular events, with most deaths occurring within the first week of hospitalization [2,3].

Hemodynamic status has significant prognostic implications; obstructive shock or refractory hypotension indicate severe right ventricular dysfunction and are associated with a high risk of early mortality, requiring prompt reperfusion therapy [3,4].

For normotensive patients with APE, representing the majority of cases, timely and precise initial prognostic assessment is pivotal in reducing early mortality and guiding selection of the most appropriate therapeutic approach. In this subgroup, therapeutic escalation decisions remain controversial, underscoring the need for improved risk discrimination. The European Society of Cardiology (ESC) guidelines recommend the use of a multiparameter risk stratification system based on clinical presentation, PESI or sPESI score, echocardiographic or CT signs of right ventricular (RV) dysfunction, and increased values of markers associated with myocardial injury and ventricular dysfunction, particularly troponin [5].

Despite its widespread adoption, the ESC algorithm for early-mortality risk stratification in APE has several inherent limitations. It relies on a multimodal assessment integrating clinical scores, RV dysfunction on imaging, and cardiac biomarkers, which may reduce feasibility in time-critical settings and introduce interobserver variability. PESI-based stratification may overestimate risk in elderly or comorbid patients, and may underestimate severity in younger individuals with significant RV impairment. The intermediate-risk category—particularly the intermediate–high-risk subgroup—remains clinically heterogeneous, so that there is limited capacity to accurately identify patients who may benefit from escalated reperfusion strategies. Moreover, imaging criteria for RV dysfunction are not fully standardized, and biomarker elevation lacks specificity.

Further refinement of early adverse event risk assessment is therefore warranted, and the integration of routinely available blood biomarkers may improve prognostic accuracy.

It is well known that inflammation and thrombosis are interdependent. Inflammation can cause endothelial damage, elevate procoagulant factors and inhibit natural anticoagulant pathways and fibrinolytic activity [6]. Inflammatory and coagulation imbalances may be reflected in hematologic abnormalities that can be detected by studying different blood cellular indices.

The neutrophil–lymphocyte ratio (NLR) is an inflammatory biomarker that combines two facets of the immune system: the innate immune response, mainly attributable to neutrophils; and acquired immunity, mediated by lymphocytes [7].

Elevated NLR has been reported in a wide range of clinical conditions, including infection [8], stroke [9], myocardial infarction [10], atherosclerosis [11], and malignancy [12], and it has been associated with increased mortality in the general population [7].

Several retrospective studies have confirmed the predictive value of NLR in assessing the risk of death among patients with APE. However, most available studies were single-center and heterogeneous, limiting the strength of evidence and its immediate clinical applicability. To address this gap, a meta-analysis of the literature was conducted in 2024, with the authors confirming the prognostic role of NLR in patients with APE and suggesting that this parameter is worth using in clinical practice [13].

Nevertheless, as these studies did not specifically address normotensive patients with APE, significant gaps and inconsistencies persist in the current understanding of the prognostic value of blood cellular indices within this risk group.

The aim of the present study was to determine whether NLR provides incremental prognostic value beyond the current ESC risk stratification model in normotensive patients with intermediate-risk APE, thereby refining early mortality risk assessment in this clinically challenging subgroup.

## 2. Materials and Methods

### 2.1. Study Design and Population

In this observational cohort study, we retrospectively analyzed the medical records of all consecutive patients diagnosed with APE and admitted to the Cardiology Department of “Sf. Pantelimon” Emergency Hospital, Bucharest, Romania, between 1 January 2014, and 31 May 2025.

The diagnosis of APE was confirmed by contrast-enhanced computed tomography pulmonary angiography (CTPA) in all cases. Baseline demographic, clinical, laboratory, and imaging data were extracted from the institutional electronic database. The Pulmonary Embolism Severity Index (PESI) score was calculated at admission using an online calculator [14].

Exclusion criteria were: absence of CTPA confirmation, missing neutrophil or lymphocyte counts at admission, age < 18 years, and hemodynamic instability. The study protocol was reviewed and approved by the Ethics Committee of “Sf. Pantelimon” Emergency Hospital (license number 49/21.08.2018) and conducted in compliance with the World Medical Association’s Declaration of Helsinki. At the time of admission, patients signed a consent form agreeing that their medical information could be used anonymously for research purposes.

### 2.2. Definitions

Hemodynamic instability was defined as: cardiac arrest, obstructive shock (systolic blood pressure (BP) < 90 mmHg or vasopressor support required to maintain a BP ≥ 90 mmHg associated with signs of hypoperfusion), or persistent hypotension (systolic BP < 90 mmHg or a systolic drop in BP ≥ 40 mmHg for >15 min, not caused by new-onset arrhythmias, hypovolemia or sepsis). Patients fulfilling these criteria were classified as high-risk according to ESC definitions and were excluded from the present analysis.

Pulmonary embolism risk classification was performed according to the 2019 ESC guidelines, with patients classified as low-, intermediate- (intermediate–low, intermediate–high), or high-risk [4]. Patients with a PESI class I–II, without RV dysfunction, were classified as low-risk. Patients with PESI class III–V were considered as intermediate-risk, and were further stratified based on imaging and biomarker findings. The presence of both RV dysfunction and elevated troponin defined the intermediate–high-risk subgroup, while patients presenting only one or none of these criteria were classified as intermediate–low-risk.

### 2.3. Biological Samples

Venous blood samples were collected in the emergency department and processed in the central laboratory. Laboratory assessment included a complete blood count, along with measurement of troponin I and NT-proBNP levels. A value above 0.2 ng/dL of serum troponin I was considered elevated and indicative of myocardial injury. A value above 125 pg/mL of serum NT-proBNP was considered elevated and indicative of ventricular dysfunction.

### 2.4. Echocardiography

Transthoracic echocardiography was performed at admission, prior to the initiation of therapy, by certified cardiologists. RV dysfunction was defined as at least one of the following parameters:

- RV dilatation in parasternal long axis view,
- RV/left ventricle ratio > 1 in the apical 4-chamber view, with end-diastolic diameters measured at the level of mitral and tricuspid valve tips in apical 4-chamber view,
- Tricuspid annulus systolic plane excursion (TAPSE) decreased to below 16 mm, as measured by M-mode in apical 4-chamber view.

### 2.5. Study Endpoint

Treatment was administered according to ESC guidelines. Most patients were treated with standard anticoagulation, either through intravenous unfractionated heparin or subcutaneous low-molecular-weight heparin. The decision to administer systemic thrombolysis with t-PA was made by the attending or on-call physician, depending on the patient’s hemodynamic condition. In-hospital all-cause mortality was the primary endpoint.

### 2.6. Statistical Analysis

Data were collected in Microsoft Excel and analyzed using Python 3.7. For database loading, processing by filtering, grouping, and calculation of descriptive statistics the pandas module [15] was used, and data visualization was performed using matplotlib [16].

The test used for group vs. group comparison was informed by the Shapiro–Wilk normality test. Normally distributed data were compared using Student’s *t* tests, while non-normally distributed data were analyzed by Mann–Whitney U tests (all from the Scipy library [17]).

Pearson coefficients were calculated (SciPy) for all pairs of variables individually, using all available paired observations, and then aggregated in a correlation matrix. The parameters were also grouped by the unweighted pair group method with arithmetic mean, and they were visualized using Seaborn version 0.13.2 [18].

In this study, the outcome of interest was in-hospital death, and both clinical and biochemical parameters were evaluated as predictors. Each parameter was assessed individually through univariate logistic regression, using a stratified train–test split (67/33%) for model fitting and sklearn for evaluation [19]. Predictive performance was quantified by accuracy, ROC curves, and optimal thresholds based on the Youden index. A multivariable logistic regression was further applied, with in-hospital death as the binary outcome, implemented in the Statsmodels library [20]. Continuous predictors used in the multivariable logistic analysis were z-score-standardized, while binary indicators were left unscaled. For each predictor, odds ratios with 95% confidence intervals were calculated. For categorical variables, the odds ratio represented the change in odds of death given the presence of the factor, while for continuous variables it reflected the change in odds associated with an increase of one standard deviation.

Throughout the document, *p* values < 0.05 were considered statistically significant.

## 3. Results

After applying the exclusion criteria, 402 normotensive APE patients were included in the study from the initial cohort of 432 subjects. Of these, 78.9% were classified in the intermediate-risk group (intermediate–high 27.5%, intermediate–low 72.5%) and 21.1% in the low-risk category.

The mean age was 67.29 ± 13.48 years. Overall, 58.0% of patients were female. The baseline clinical and laboratory characteristics are represented in Table 1 and Table 2. In-hospital mortality was 13.97% across all analyzed groups. The median duration of hospitalization was 9 days (2–20).

Patients who died during hospitalization were older, had a higher prevalence of neoplasia, and, as expected, exhibited higher PESI scores.

Laboratory assessment revealed significant increases in neutrophil counts and in NLR, lactate, and NT-proBNP levels in patients with in-hospital mortality. Lymphocyte levels were lower in non-survivor patients.

### 3.1. Assessing the Influence of Demographics and Comorbidities on NLR Value

The median NLR value in our cohort was 4.86 (25–75%: 2.86–8), with no significant differences between sexes. A weak but statistically significant positive correlation was observed between NLR and patient age (*p* = 0.0029, Pearson R: 0.149) and with elevated heart rate (*p* = 0.0031, Pearson R: 0.148).

A higher NLR value was identified in patients with APE and associated neoplasia (7.63 ± 5.9 vs. 6.4 ± 7.09, *p* = 0.004), but this remained independently associated with higher mortality. A total of eighty-one patients with neoplasia and associated APE were analyzed; of these, seventy-eight had solid tumors and three had hematological malignancies.

### 3.2. Correlation of NLR Value with RV Dysfunction, NT-ProBNP, Troponin and Other Biological Parameters

No significant association was observed between NLR and troponin I value (*p* = 0.57, Pearson R = 0.02) or with the TAPSE echocardiographic parameter (*p* = 0.17, Pearson R = −0.07). However, there was a statistically significant positive correlation with NT proBNP value (*p* = 0.0001, Pearson R = 0.25) and serum creatinine (*p* < 0.0001, Pearson R = 0.2).

### 3.3. Value of NLR in Predicting In-Hospital Mortality

The NLR value was significantly higher in patients who died during hospitalization (*p* < 0.0001), associated with an acceptable AUC of 0.650, regardless of the presence of neoplasia. The cut-off level identified by the Youden’s index-associated criterion was 5.49 (76.5% sensitivity, 54.4% specificity). This was the optimal threshold used in our study to analyze the added value of NLR on top of the 2019 ESC Algorithm.

**Figure 1:**
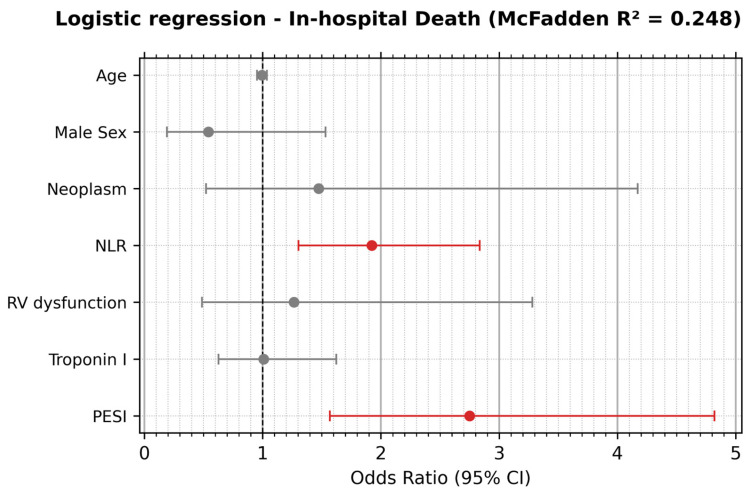
Forest plot showing odds ratios and 95% intervals. Each reported odds ratio (OR) for a continuous variable reflects the multiplicative change in the odds of in-hospital death per +1 SD increase in that variable. Binary-variable ORs compare the indicated category to its reference (RV dysfunction present vs. absent). SDs used for standardization of significant predictors were: NLR = 5.66, PESI = 29.67. N = 291 patients (due to missing data). In red lines we present variables that are independently associated with in-hospital mortality.

In the multivariable model adjusted for significant covariates, NLR remained independently associated with in-hospital mortality, with values exceeding one standard deviation (5.66) indicating an approximately twofold higher risk of in-hospital mortality, whereas RV dysfunction and troponin I did not retain independent prognostic significance (Figure 1).

### 3.4. Value of NLR in Combination with Validated Risk Scores—PESI and 2019 ESC Algorithm

The accuracy of the PESI score in correctly classifying patients into risk categories was 46.2%, considering the identification of patients with intermediate risk APE (from class III and above).

#### 3.4.1. Intermediate–High-Risk APE Patients

The 2019 ESC algorithm (PESI score, presence of RV dysfunction, and elevated troponin I) correlated with an accuracy of 76.5% in correctly identifying patients with intermediate–high-risk APE. Adding NLR above a value of 5.49 to the 2019 ESC Algorithm resulted in the best accuracy (85.2%) among the parameters studied for correctly identifying patients with APE and intermediate–high risk (Table 3).

#### 3.4.2. Intermediate–Low-Risk APE Patients

Following the analysis performed on our cohort, it was observed that the in-hospital all-mortality rate of these patients was 14.3%. Among intermediate–low-risk patients, those with NLR > 5.49 had a sixfold higher in-hospital mortality (29.4% vs. 4.9%). As a result, the addition of NLR assessment for normotensive PE patients at intermediate–low risk identified those at risk of early death with a high sensitivity of 78%, allowing them to be reclassified into a higher risk group (Table 4).

## 4. Discussion

In our cohort of normotensive patients with APE, the NLR emerged as an independent predictor of in-hospital mortality. Notably, NLR demonstrated incremental prognostic value within the heterogeneous intermediate-risk category, where early identification of patients at higher risk remains clinically challenging. While its overall discriminative ability was modest, the addition of NLR to established risk stratification tools improved the identification of patients at increased risk of adverse in-hospital outcomes.

Normal NLR values remain a subject of debate in the current literature. Forget et al. [21], in a retrospective study, observed that normal NLR values in an apparently healthy non-geriatric adult population can range from 0.78 to 3.53. In another study published in 2018 [22], a mean NLR of 1.76 (0.83–3.92) was reported for a general population. The median NLR value of our patients diagnosed with APE was 4.85 (CI 5–95%: 1.42–17.74), significantly higher than the normal values reported in previous studies.

In our analysis, the NLR value was not influenced by the gender of the patients, although a previously published study suggested that the average NLR value was significantly higher in men [22]. Advanced age was associated with higher NLR values, a finding consistent with previous reports demonstrating a positive correlation between age and systemic inflammatory markers such as NLR [23].

A NLR value above 5.49 was associated with the best specificity and sensitivity for detecting the risk of in-hospital death. This value is relatively similar to that described by Efros et al. in 2021 [24], so we propose using this threshold to refine the detection of early mortality risk. This consistency across diverse populations supports its potential reproducibility and clinical applicability as a prognostic marker.

Several prior investigations have reported an association between elevated NLR and adverse outcomes in APE. However, most studies included hemodynamically unstable patients or did not focus specifically on normotensive individuals. By restricting our analysis to normotensive patients, we addressed a clinically relevant subgroup in which risk stratification is more nuanced and therapeutic decisions are less straightforward. Our findings support previous evidence suggesting that inflammatory biomarkers may complement conventional prognostic indicators, while highlighting their potential utility specifically within intermediate-risk populations.

Interestingly, NLR was not associated with RV dysfunction or increased troponin I, which may indicate another facet of this pathology, namely, vascular inflammation, reflecting an immune imbalance associated with worse outcomes. A study published in 2017 suggests that early inflammation may induce endothelial dysfunction in APE patients, which may explain why the prognosis for these patients is less favorable [25].

The homeostasis between these two aspects of the immune response represented in the NLR value is often fragile. This balance could be affected by multiple pathological factors such as the increased activity of the neurohormonal and adrenergic systems, secondary to severe hypoxia as a result of pulmonary artery obstruction, which could induce the release of inflammatory cytokines. High levels of these substances are associated with an increase in the number of neutrophils and a simultaneous reduction in the number of lymphocytes. Such a response could exacerbate thrombosis and influence the clinical condition of patients [26].

Risk stratification in normotensive APE remains imperfect, particularly within the intermediate-risk category, where clinical trajectories can vary considerably. Although echocardiographic right ventricular dysfunction and cardiac biomarkers are integral to current guidelines, these did not retain independent prognostic significance in our adjusted model. In this context, NLR may represent a simple, inexpensive, and widely available adjunctive biomarker that can be obtained at admission without additional cost or delay. Its incorporation into existing algorithms may aid in refining early prognostic assessment, especially when conventional parameters yield borderline or inconclusive results.

However, given the modest discriminative capacity observed, NLR should be viewed as complementary to established risk assessment tools, rather than as substitutive.

We acknowledge some limitations to our study. Particularly, we highlight the retrospective application of the scores as well as the relatively small number of patients included at a single center. The included patients were diagnosed and treated over a long period of time, which may limit the reliability of the present results.

Our findings align with this body of evidence, reinforcing NLR’s role as an accessible, cost-effective tool that may enhance the discriminative power of existing risk stratification algorithms, and enable more personalized therapeutic decision-making for normotensive APE patients.

We propose adding admission NLR (>5.49) as a simple adjunct to the ESC 2019 algorithm to upwardly reclassify intermediate-risk normotensive APE patients for intensified monitoring and earlier multidisciplinary consideration of reperfusion; decisions on reperfusion remain based on composite clinical, imaging and biomarker trends.

## 5. Conclusions

In our study, NLR was independently associated with in-hospital mortality in normotensive patients with APE. An NLR threshold > 5.49 may provide additional prognostic information when integrated into the 2019 ESC risk stratification algorithm, potentially improving the identification of patients at increased risk of early death within both intermediate–high and intermediate–low risk categories. Prospective validation is warranted before routine clinical implementation.

## Figures and Tables

**Table 1:** Clinical data table.

Parameter	Lived	Died	All	*p*-Value ^#^
Number	346	56	402	
Sex				
Male	148 (42.8%)	21 (37.5%)	169 (42.0%)	0.551
Female	198 (57.2%)	35 (62.5%)	233 (58.0%)	
Age (years)	66.5 ± 13.4	72.5 ± 12.9	67.3 ± 13.5	**0.002**
18–35	5 (1.4%)	0 (0.0%)	5 (1.2%)	**0.006**
35–65	125 (36.1%)	16 (28.6%)	141 (35.1%)	
65–80	160 (46.2%)	20 (35.7%)	180 (44.8%)	
80–100	56 (16.2%)	20 (35.7%)	76 (18.9%)	
Neoplasia	62 (17.9%)	19 (33.9%)	81 (20.1%)	**0.011**
Dyspnea	271 (78.3%)	46 (82.1%)	317 (78.9%)	0.636
Syncope	43 (12.4%)	9 (16.1%)	52 (12.9%)	0.595
Leg edema	98 (28.3%)	11 (19.6%)	109 (27.1%)	0.27
Chest pain	115 (33.2%)	3 (5.4%)	118 (29.4%)	**<0.001**
Shock Index	0.8 ± 0.2	0.9 ± 0.3	0.8 ± 0.2	**<0.001**
Modified Shock Index	1.0 ± 0.3	1.2 ± 0.4	1.1 ± 0.3	**<0.001**
PESI	98.6 ± 28.4	136.3 ± 37.1	103.9 ± 32.5	**<0.001**

^#^ The distribution of categorical variables (sex, age bins, neoplasia, dyspnea, syncope, leg edema and chest pain) was assessed by chi-square tests; all other *p*-values shown represent values obtained in Student’s *t* tests. Statistically significant *p*-values are shown in bold. PESI = Pulmonary Embolism Severity Index.

**Table 2:** Comparison of laboratory parameter values between the survival groups.

Variable	Lived		Died		*p*-Value ^#^
	Median	25–75%	Median	25–75%	
NLR	4.4	2.78–6.87	8.19	5.11–14.22	**<0.0001**
Lymphocytes	1810	1307.5–2500	1210	645–1995	**<0.0001**
Lactic acid	17.15	12.17–22.92	20.315	15.42–40.28	**0.0021**
Neutrophils	8005	6280–10,492.5	10,710	6755.5–14,122	**0.0022**
NT PRO BNP	1587.5	359.5–4115	3742	1110–9649	**0.0023**
Leukocytes	11,220	8950–14,050	13,355	9600–18,966	**0.0146**
PH	7.44	7.4–7.48	7.43	7.3–7.47	**0.015**
Hb	13.6	12.2–14.9	13.1	11.16–14.33	**0.0237**
Na	139	136–142	138	133–142	0.1096
PaCO_2_	29.2	26–32.6	30.4	26.15–37.57	0.3124
PaO_2_	62.5	52.27–76.05	64.3	49.4–89.7	0.4175
Creatinine	0.96	0.77–1.13	1	0.77–1.3	0.4488
Thrombocytes	228,000	180,000–288,000	219,500	141,500–299,000	0.5005
Troponin I	0.227	0.2–0.81	0.255	0.2–0.94	0.6428
K	4.22	3.9–4.62	4.39	3.82–4.72	0.8352

^#^ *p*-values obtained using Mann–Whitney U test, except in the case of potassium where normality was satisfied for both data groups and Student’s *t* test was used. Statistically significant *p*-values are shown in bold.

**Table 3:** Sensitivity, specificity, and accuracy of selected parameters for the identification of intermediate–high-risk APE patients.

Feature	N	tp (%)	tn (%)	fp (%)	fn (%)	Sensitivity	Specificity	Accuracy
NLR eval	398	9.5	54.3	31.9	4.3	69.1	63	63.8
PESI eval	398	13.3	32.9	53	0.8	94.6	38.3	46.2
PESI-NLR score	395	11.9	58.2	27.8	2	85.5	67.6	70.1
2019 ESC algorithm	293	2.4	74.1	16	7.5	24.1	82.2	76.5
2019 ESC algorithm plus NLR	291	2.1	83.2	7.2	7.6	21.4	92	85.2
NLR AND PESI	395	9.4	64.8	21.3	4.6	67.3	75.3	74.2

PESI eval = PESI > 86, PESI-NLR score =PESI + 3.76∙NLR, 2019 ESC algorithm = PESI > 86 AND troponine > 0.2 AND RV dysfunction, 2019 ESC algorithm plus NLR = PESI > 86 AND troponine > 0.2 AND RV dysfunction AND NLR > 5.49, NLR AND PESI = PESI > 86 AND NLR > 5.49.

**Table 4:** Sensitivity, specificity, and accuracy of selected parameters for the identification of intermediate–low-risk APE patients.

	N	tp (%)	tn (%)	fp (%)	fn (%)	Sensitivity	Specificity	Accuracy
NLR eval in High PESI, low troponin, no RV dysfunction	57	8.8	59.6	26.3	5.3	0.625	0.694	0.684
NLR eval in High PESI, low troponin, RV dysfunction	22	13.6	63.6	18.2	4.5	0.75	0.778	0.773
NLR eval in High PESI, high troponin, no dysfunction	54	13	55.6	31.5	0	1	0.638	0.685
NLR eval in all above	133	11.3	58.6	27.1	3	0.789	0.684	0.699

High PESI > 86, NLR > 5.49.

## Data Availability

All data is provided within the article.

## Abbreviations

The following abbreviations are used in this manuscript:

APE	Acute pulmonary embolism
RV	Right ventricle
NLR	Neutrophil–lymphocyte ratio
